# *BrRCO* promotes leaf lobe formation by repressing *BrACP5* expression in *Brassica rapa*

**DOI:** 10.1093/hr/uhaf084

**Published:** 2025-03-12

**Authors:** Yunxia Sun, Limin Hu, Junrey C Amas, William J W Thomas, Lihui Wang, Xian Wang, Wei Wang, Gaoyang Qu, Xiaoxiao Shen, Ruiqin Ji, Jacqueline Batley, Chuchuan Fan, Yugang Wang

**Affiliations:** Department of Horticulture, Shenyang Agricultural University, Shenyang 110866, China; Key Laboratory of Protected Horticulture, Ministry of Education, Shenyang Agricultural University, Shenyang 110866, China; National Key Laboratory of Crop Genetic Improvement, Huazhong Agricultural University, Wuhan 430070, China; National Key Laboratory of Smart Farm Technology and System, Northeast Agricultural University, Harbin 150006, China; School of Biological Sciences and the Institute of Agriculture, The University of Western Australia, Crawley, WA, 6009 Australia; School of Biological Sciences and the Institute of Agriculture, The University of Western Australia, Crawley, WA, 6009 Australia; Department of Horticulture, Shenyang Agricultural University, Shenyang 110866, China; Department of Horticulture, Shenyang Agricultural University, Shenyang 110866, China; Department of Horticulture, Shenyang Agricultural University, Shenyang 110866, China; Department of Horticulture, Shenyang Agricultural University, Shenyang 110866, China; Key Laboratory of Protected Horticulture, Ministry of Education, Shenyang Agricultural University, Shenyang 110866, China; National Key Laboratory of Crop Genetic Improvement, Huazhong Agricultural University, Wuhan 430070, China; Department of Horticulture, Shenyang Agricultural University, Shenyang 110866, China; School of Biological Sciences and the Institute of Agriculture, The University of Western Australia, Crawley, WA, 6009 Australia; National Key Laboratory of Crop Genetic Improvement, Huazhong Agricultural University, Wuhan 430070, China; Department of Horticulture, Shenyang Agricultural University, Shenyang 110866, China; Key Laboratory of Protected Horticulture, Ministry of Education, Shenyang Agricultural University, Shenyang 110866, China; Hainan Seed Industry Laboratory, Sanya 572025, China

## Abstract

Lobed leaves are advantageous for gas exchange, canopy architecture, and high-density planting; however, the genetic mechanisms of leaf lobe formation in *Brassica* crops remains poorly understood. Here, *lob10.1*, our previously identified major QTL controlling the presence/absence of leaf lobes in *B. rapa* (AA), was fine mapped to a confidence interval of 69.8 kb. *REDUCED COMPLEXITY ORGAN* (*BrRCO*, *BraA10g032440.3c*), a homeodomain leucine zipper class I (HD ZIP I) transcription factor, was predicted to be the most likely candidate gene underlying *lob10.1*. Null mutations of *BrRCO* by CRISPR/Cas9 in the lobed-leaf parent RcBr and over-expression in the counter-part near isogenic lines (NIL^RcBr^) lead to entire and lobed leaves, respectively. Analysis of the gene evolution revealed that *A10. RCO* functions as a core gene and was generally negatively selected in *B. rapa*. Moreover, *BrRCO* function as a negative regulator by directly binding to promoters of *BrACP5* and repressing its expression. The function of *ACID PHOSPHATASE TYPE 5* (*BrACP5*) was subsequently confirmed as VIGS-BrACP5 produced entire leaves in RcBr. This study identified the core gene *BrRCO* to be involved in the development of leaf lobes in *B. rapa* and elucidated a new pathway for leaf lobe formation by the BrRCO-*BrACP5* module. These findings provide a theoretical basis for the formation of leaf lobes in *Brassica* crops.

## Introduction

The shape of leaves can be either entire or elaborated to form serrations or lobes, of which there are a wide range of natural variations in *Brassica* germplasm [1]. Leaf margin morphology is frequently used to discriminate plant species, which have adapted to the environment and have evolved through long-term natural and artificial selection [2]. Some studies have indicated that lobed leaves show advantages in plant stress resistance, including strong winds, standing drought, and salinity [3–5]. Furthermore, the varieties with lobed leaves have shown potential advantages in high-density planting for mechanized agriculture, and are therefore beneficial to *Brassica* improvement [6]. Thus, it is of great biological and economic importance to unravel the molecular mechanism of leaf lobe formation in *Brassica* crops.

To date, a number of genes playing a central role in determining leaf shape formation have been identified in diverse species. For example, *CUP-SHAPED COTYLEDON2* (*CUC2*), *PIN-FORMED 1* (*PIN1*), and *miR164* function by regulating auxin activity, which determines the position of the serrations and leaflets in *Arabidopsis thaliana* [7, 8]. Class I *KNOTTED1-LIKE HOMEOBOX* (*KNOX1*) is also a requirement for leaflet development, where no expression in *A. thaliana* produces deep lobes [9]. In *A. thaliana*, the HD-ZIP I gene family *LATE MERISTEM IDENTITY1* (*LMI1*) and *LEAFY* (*LFY*) co-activate *CAULIFLOWER* (*CAL*) expression, or *LFY*-independent, to promote the formation of simple leaves with serrated margins [10]. *LMI1-like* genes have been predicted as the most promising candidate genes underpinning lobed leaves in watermelon and upland cotton; however, the functionality has only been validated in cotton using virus-induced gene silencing (VIGS) [11, 12]. *RCO*, arose by duplication of the genes of its ancestral paralog, *LMI*, which is lost in *A. thaliana*, resulting in a simplified leaf in *Brassica* species [13].

In *Brassica* crops, there has been a major focus on genetics and gene mapping for leaf lobe formation. On chromosome A10, a locus with major effects controlling leaf lobes has been consistently found in *B. rapa* [14–16], *B. napus* [5, 17–19], and *B. juncea* [20–22], indicating that this region at the end of chromosome A10 should be a hot spot region controlling leaf lobe formation in *Brassica* species. Recently, in *B. rapa*, the regulation of leaf lobe formation by *BrLMI1* was functionally validated by ectopic expression in *A. thaliana* [16]. Our previous studies showed that *BnA10. RCO* and *BnA10. LMI* can function separately or together to determine leaf lobes formation in *B. napus* [18, 19]. Variations in the promoter of *BrLMI1*, as well as *BnA10. LMI* in *B. napus* [18], are likely to be causal in determining leaf lobe formation [16]. In *B. juncea*, *BjA10. RCO* was also predicted as the most promising causal gene responsible for leaf lobe formation [20, 22], which was further verified by CRISPR/Cas9-based editing in leaf mustard XC [21]. Thus, whether *A10. RCO* plays a key role in leaf lobe formation in *B. rapa* remains an intriguing question.

The development of leaf lobe involves combined processes including microRNAs, transcriptional regulators, and phytohormones. For example, the local auxin activity determines the origin of leaf primordia [23]. Besides auxin, leaf initiation and development also require cytokinins and ethylene response factors [24]. A previous study showed that *RCO* inhibits the growth of developing leaflet bases, thus highlighting the differences in growth resulting from edge patterning [25], and controls growth by orchestrating the homeostasis of the cytokinin pathway in *Cardamine hirsuta* [26]. One study in *Arabidopsis* showed the involvement of cytokinin signaling in nutrient sensing and the significance of phosphorus-starvation signaling [27]. Nevertheless, the *RCO* target genes and molecular mechanisms are not fully understood in a variety of plants, including the *Brassica* species.

In our preliminary work, a major QTL, *lob10.1*, controlling leaf lobes in *B. rapa* has been validated [15]. In this study, NIL^RcBr^-F_2_ and F_2:3_ populations of *B. rapa* were constructed*,* and *lob10.1* was finally narrowed down to a physical distance of 69.8 kb at the end of chromosome A10. We predicted that the HD-ZIP I transcription factor *BrRCO* was the most promising candidate gene underlying *lob10.1*. The function of the candidate gene was verified by both CRISPR/Cas9 gene editing and over-expression in *B. rapa*. Gene evolution analysis for *A10. RCO* was conducted using three pangenomes containing 71 *B. rapa*, 76 *B. napus*, and 86 *B. oleracea* accessions, respectively [28]. Combining RNA-seq with DNA affinity purification sequencing (DAP-seq), the possible target genes of *BrRCO* were explored. Our results provide a strong foundation for clarifying the molecular mechanism of leaf lobe formation in *B. rapa* and also provide a theoretical reference for enhancing leaf margin characteristics in related crops.

## Results

### Narrowing down the confidence interval controlling leaf lobes in *B. rapa*

**Figure 1:**
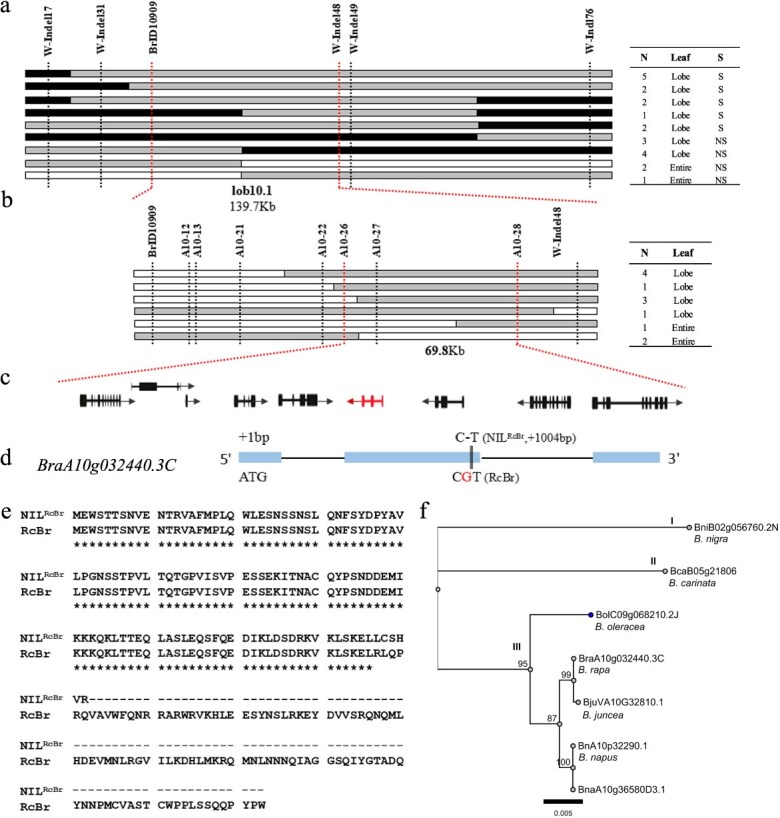
Map-based cloning of genes underlying the *lob10.1* locus. (a and b) Graphical genotyping demonstrating fine-mapping leaf lobe (black rectangle) and entire leaf (white rectangle) morphology using RcBr and Near-isogenic lines (NIL^RcBr^), along with the mapping interval. Primary genotyping and phenotyping in F_2_ populations with 22 recombinant families (a) and F_2:3_ populations with 12 recombinants (b) allow delimiting the *lob10.1* locus into a region of ~139.7 and ~ 69.8 kb, respectively. N: Number, S: Segregation and NS: No segregation. (c) Annotated genes in the confidence interval between the A10–26 and A10–28 markers. The arrows indicate the orientation of the genes, the black boxes represent the coding regions and the candidate gene is marked with a triangle. (d) There is a G-base insert in RcBr shown by a vertical line, detected in the second exon of *BrRCO*^RcBr^. (e) The protein prediction indicated that the gene was terminated early in translation in NIL^RcBr^. (f) Analysis of Phylogenetic tree.

Previously, we validated the major QTL, *lob10.1*, controlling the presence/absence of leaf lobes*,* in *B. rapa* with a set of NIL^RcBr^, developed with two Insertion–Deletion (InDel) markers, BrID10909 and BrID10233 [15]. To identify the candidate genes controlling leaf lobe development, a preliminary marker analysis was undertaken using a NIL^RcBr^-F_2_ segregating population, which was derived from a cross between RcBr and NIL^RcBr^. Then, *lob10.1* was mapped to a 139.7 Kb confidence interval, flanked by the BrID10909 and W-InDel48 markers (Fig. 1a). Then, single nucleotide polymorphism (SNP) and InDel markers were designed depending on sequences variations in the confidence interval between the two parental lines, NIL^RcBr^ and RcBr (Table S1). In the first instance, two SNP markers were utilized to screen 4000 F_2:3_ individuals derived from the NIL^RcBr^-F_2_ populations, and 35 recombinant monocultures were screened by kompetitive allele-specific PCR (KASP) genotyping. Seven polymorphic markers were then utilized to screen the recombinant monocultures by polypropylene gel electrophoresis (PGE). Finally, the confidence interval was narrowed down to a physical distance of 69.8 Kb (20 051 392-20 121 184) at the end of chromosome A10, between A10–26 and A10–28 markers (Figs 1b and Fig. S1).

### *BrRCO* is the most promising candidate gene underlying *lob10.1* in *B. rapa*

A total of nine genes were detected in the confidence interval of *lob10.1* in *B. rapa* (Fig. 1c). The functions of the nine genes were predicted by searching for corresponding putative orthologous genes in the *Arabidopsis* annotation database (https://www.arabidopsis.org/, Table S2). Six genes were found to have sequence differences in the coding regions and three out of six (*BraA10g032430.3C*, *BraA10g032440.3C,* and *BraA10g032470.3C*) showed sequence variations in the predicted proteins between the two parental lines, RcBr and NIL^RcBr^. Of these, only *BrRCO*, which encodes an HD-Zip I transcription factor, which is homologous to *AtLMI1 (AT5G03790),* showed a significant difference in expression from RNA-seq analysis (Table S3).

We also cloned the full-length gDNA and cDNA sequences of *BrLMI* (*BraA10g032450.3c*) and *BrRCO* in the two parental lines, RcBr and NIL^RcBr^, respectively. *BrRCO*, consisted of three exons and two introns, and a G-insertion was detected in the second exon of *BrRCO*^RcBr^ compared with NIL^RcBr^ (+1004 bp from initiating codon), while no sequence variations were detected for *BrLMI* between the two parents (Fig. 1d-e; Figs S2-S5). As such, *BrRCO* was selected as the most likely candidate gene for *lob10.1* in *B. rapa*.

### Selection and phylogenetic analysis of *BraA10g032440.3C*

The search in three *Brassica* pangenomes [28] identified *BnaA10g36580D3* as the homologous gene of *BraA10g032440.3C* in *B. napus* with 98.60% pairwise identity (Table S4). This gene is present in all the *B. napus* (72) and *B. rapa* (71) lines that were included in the pangenome and is therefore considered a core gene in these two species (Table S5). However, in the *B. oleracea* pangenome, this gene was only found in 46 out of the 86 lines and was classified as a variable gene in *B. oleracea*.

The dN/dS values of *BraA10g032440.3C* relative to its homologs in the other *Brassica* species (*B. napus*, *B. juncea*, *B. nigra,* and *B. oleracea*) were less than one, indicating this gene is generally negatively selected in these species (Table S6). However, when compared to *B. carinata,* the dN/dS ratio was more than one, indicating this gene is positively selected in this species. Moreover, phylogenetic analysis revealed that *B. nigra* (*BB*) and *B. carinata* (*BBCC*) homologs were separated from the rest of the species (Fig. 1f). *BraA10g032440.3C* was clustered along with its *B. juncea*, *B. napus,* and pangenome homologs.

### Functional verification of *BrRCO* in *B. rapa*

**Figure 2:**
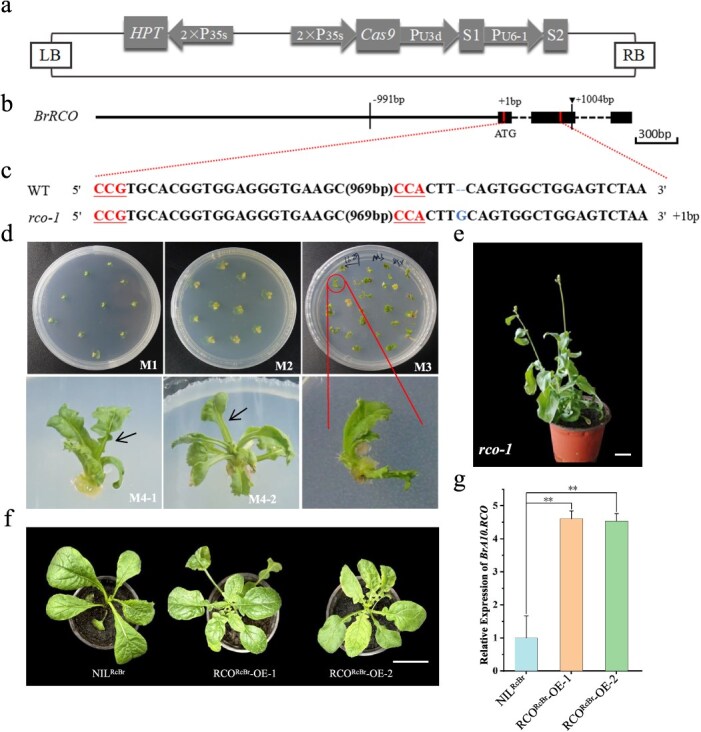
Functional verification of *BrRCO* in *B. rapa*. (a) Binary constructs of Cas9P35s-RCO. (b) Gene structure of *BrRCO* and natural variations between the alleles from NIL^RcBr^ and RcBr. Down arrow indicates base insertion in RcBr, black vertical line indicates SNP. Black boxs indicate exons, and the two vertical lines on the exons indicate the positions of CRISPR/Cas9 sgRNA. (c) Illustration of the InDel patterns of Cas9P35s-RCO-edited RcBr mutants. The target sequences are shown with the protospacer-adjacent motif (PAM) with the underlined. WT: wild type; *rco-1*: T_0_ mutant. (d) The flow of the *Agrobacterium* stem cotyledon transformation method. (e) Phenotypes of mutant T_0_ plants. Bars = 5 cm. (f) Phenotypes of overexpression *BrRCO*^RcBr^ in NIL^RcBr^. Bars = 5 cm. (g) Expression comparison between NIL^RcBr^ and two overexpression lines.

In our previous study, a CRISPR/Cas9 construct was generated. The construct contained two sgRNAs within *BnA10.RCO* with Cas9 driven by *Cauliflower mosaic virus* (CaMV) 35S, i.e. SBnRCO [19] (Fig. 2a), and those two sgRNAs exactly target *BrRCO* (Fig. 2b).

In *B. rapa*, we obtained a homozygous *rco* knockout line *rco-1*, carrying a G-base insertion, which resulted in loss of function of the BrRCO protein (Fig. 2c-d), and the *rco-1* seedlings had an entire-leaf phenotype (Fig. 2e). We examined the temporal dynamics of subcellular localization of *BrRCO* in *B. rapa*, and the fluorescence signal overlapped with the signal of the nucleus marker, which is consistent with the function of *LMI1-*like genes as transcription factors (Fig. S6). We also developed transgenic lines that overexpressed *BrRCO*^RcBr^ under NIL^RcBr^ background. As expected, NIL^RcBr^ overexpression lines produced deep lobes and had significantly higher expression compared to the wild type (WT), NIL^RcBr^ (Fig. 2f-g).

**Figure 3:**
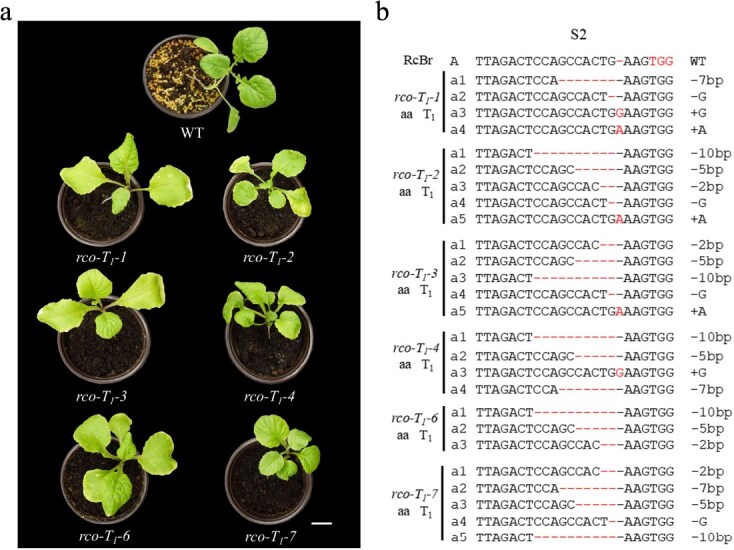
*BrRCO* mutant genotypes and phenotypes of Cas9P35s-RCO-edited T_1_ plants. (a) T_1_ generation seedling phenotype. WT with lobed leaf margins (RcBr), T_1_ generation plants with entire leaf margins (*rco*-T_1_–1 ~ 7). Bars = 3 cm. (b) Sequences at the sgRNA target sites of Cas9P35s-RCO-edited T_1_ plants. The nucleotide InDels are marked with horizontal lines, with details labeled at right; ‘A’ represents the WT allele, while ‘a’ represents the mutated allele.

In the T_1_ and T_2_  *rco* knockout lines, this variant trait was stably inherited. Compared to the WT RcBr, T_1_ lines of the six phenotypically variable plants (*rco-T_1_–1 ~ 8*, regrettably, the *rco-T_1_–5* and *rco-T_1_–8* seedlings did not survive) and all T_2_ lines showed entire (non-lobed) leaves (Figs 3a and S7a). The mutations in all T_1_ transgenic plants as well as T_2_ generation mutants were identified through high-throughput tracking of mutations (Hi-TOM) sequencing analysis, unlike the T_0_ line, many mutant sequences were obtained (Figs 3b, S7b and Table S7). Most of the T_2_ generation of transgenic lines showed different types of mutant sequences and were positive for the CRISPR/Cas9 transgene (Fig. S7c). The *rco*-7-2 transgenic plant, which is negative for the CRISPR/Cas9 transgene and has a single type of mutant sequence, was used for further analysis.

### Identification of potential target genes for *BrRCO*

In order to predict the potential gene interaction networks of *BrRCO*^RcBr^ in leaf lobe formation, the expression networks identified for the DEGs between NIL^RcBr^ vs. RcBr and *rco-7-2* vs. RcBr were constructed, respectively. Accordingly, 189 up-regulated and 94 down-regulated DEGs were identified between NIL^RcBr^ vs. RcBr, while 279 up-regulated and 311 down-regulated DEGs were identified between *rco-7-2* vs. RcBr, including nine up-regulated genes and six down-regulated genes collectively (Fig. S8a). Twelve out of the 15 overlapping DEGs were functionally annotated. Furthermore, excluding up-down-regulation of DEGs, there were 35 overlapping genes in the two RNA-seqs, and 34 DEGs were functionally annotated. These genes are mainly involved in processes such as polysaccharide biosynthesis, cellular polysaccharide biosynthesis, polysaccharide metabolism, and cellular carbohydrate biosynthesis. The KEGG pathway was primarily enriched in glucosinolate biosynthesis (Fig. S8b-c).

**Figure 4:**
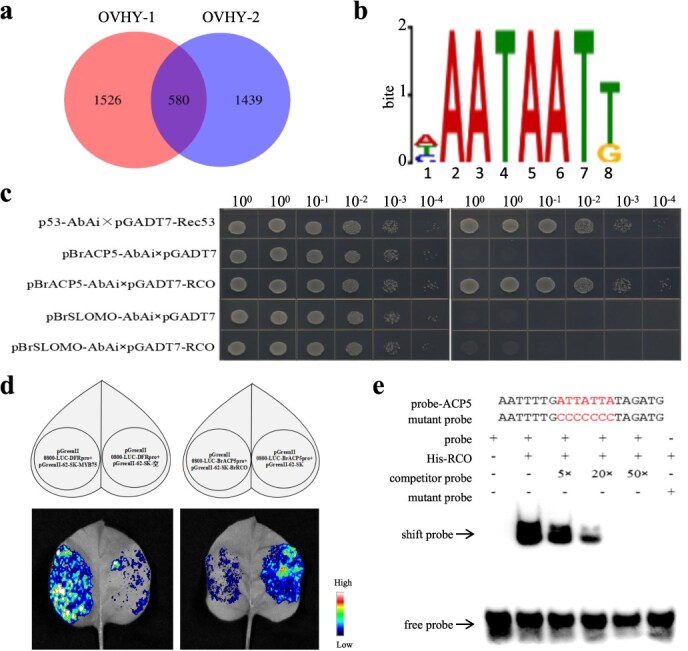
Identification of genome-wide direct targets of BrRCO. Venn diagram of peaks merged. The size of the circle indicates the number of peaks in the sample. (b) Binding motif of *BrRCO*^RcBr^ by DAP-seq. The higher the letter, the more conservative it is. (c) Y1H analysis of BrRCO directly targeting the *BrACP5* and *BrSLOMO* promoter fragment. The prey vector, pGADT7-RCO; the bait vector, p*BrACP5*-pAbAi and p*BrSLOMO*-pAbAi. Positive control, pGADT-Rec53; negative control, p*BrACP5*-AbAi and p*BrSLOMO*—AbAi. (d) DLRA of the interaction between BrRCO and the promoters of *BrACP5* genes. A schematic of transient co-expression of effectors and reporters is at the top; an image of luciferase activity is at the bottom. pGREEN-62-SK + pGREEN-0800-LUC-DFRpro indicates transient co-expression of empty effector (without BrRCO) and empty reporter (without promoter); pGREEN-62-SK-MYB75 + pGREEN-0800-LUC-DFRpro indicates transient co-expression of effector and empty reporter; pGREEN-62-SK + pGREEN-0800-LUC-BrACP5pro indicates transient co-expression of empty effector and reporter; and pGREEN-0800-LUC-BrACP5pro + PtoWRKY62-SK-RCO indicates co-expression of effector and reporter. (e) EMSA showing BrRCO the binding of *BrACP5*.

To explore the direct targets of *BrRCO*^RcBr^, DAP-seq was conducted [29]. A total of 1526 and 1439 notable peaks were identified in the genomic regions of the two technical replicates, respectively (Fig. 4a). The MEME suite was utilized to identify the core sequence and motif of (A/T/C) AATAAT (T/G) (Fig. 4b). GO pathway enrichment analysis revealed that the target genes of *BrRCO*^RcBr^ were primarily enriched in cellular metabolic process, and KEGG pathway was primarily enriched in biosynthesis of secondary metabolites and metabolic pathways (Fig. S9).

We prioritized two genes (*BraA05g029790.3C*, *BraA08g016720.3C*) co-expressed with RcBr, were found to be differentially expressed in NIL^RcBr^ vs. *rco-1* (Table S8). *BrACP5*, which is phosphate-responsive in both roots and shoots [30]; *BrSLOMO* (*SLOW MOTION*) encodes *SLOMO*, and is necessary for maintaining auxin homeostasis and for the timely initiation of lateral organs at the shoot meristem [31].

### BrRCO directly regulated *BrACP5* expression to regulate leaf lobe formation

To verify whether BrRCO directly targeted the promoter sequence of *BrACP5* or *BrSLOMO*, we introduced the promoter fragment of the three genes into yeast 1-hybrids (Y1H). This revealed that BrRCO specifically targets the promoter of *BrACP5* (Fig. 4c). Then, we carried out luciferase complementation (LUC) assay and electrophoretic mobility shift assay (EMSA) interaction experiments. The results showed that BrRCO directly targeted the promoter of *BrACP5* and inhibited the transcription level of *BrACP5 in vivo* (Figs 4d-e and S10). However, *BrACP5* expression was downregulated in both NIL^RcBr^ and *rco-7-2* (Fig. S11).

### *BrACP5* affects the phosphate-responsive pathway regulating leaf lobes formation in *B. rapa*

**Figure 5:**
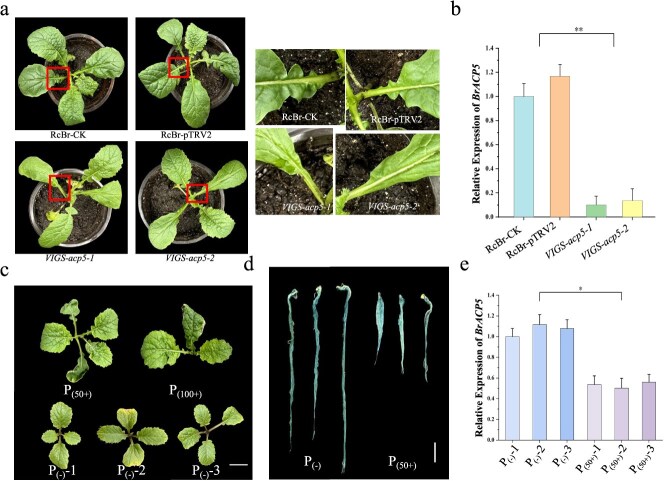
BrRCO negatively regulated *BrACP5* to regulate leaf lobe formation. (a-b) VIGS-acp5 system within RcBr. qRT-PCR analysis of *BrACP5* expression in VIGS system (a) and the phenotype of VIGS-acp5 plant. Lobe of first two true leaves mutating into rounded leaves (b). (c) Phenotype of RcBr under P_(50+)_, P_(100+)_ and P_(−)_ conditions. Bars = 2 cm. (d) Root-associated APase activity in the RcBr background under P_(−)_ and P_(50+)_ conditions. Bars = 2 cm. (e) qRT-PCR analysis of *BrACP5* expression under P_(−)_ and P_(50+)_ conditions, with three biological replicates. P_(50+)_: Hoagland’s culture solution with 50 mg/L phosphorus; P_(100+)_: Hoagland’s culture solution with 100 mg/L phosphorus; P_(−)_: Hoagland’s culture solution with no extra phosphorus.

To test whether *BrACP5* was also associated with leaf lobe formation, mutant *VIGS-acp5* were generated using the VIGS system in RcBr. Accordingly, lower levels of *BrACP5* expression were detected in *VIGS-acp5* plants, and the *VIGS-acp5* plants exhibited loss of leaf lobes compared to RcBr (Fig. 5a and b). Therefore, we proposed that leaf lobe formation was also influenced by BrACP5 protein levels.

To verify whether leaf cleavage is associated with the phosphate-responsive pathway, RcBr was tested under hydroponic conditions. The results showed that under phosphorus-deficient conditions, RcBr showed an entire-leaf phenotype (Fig. 5c). 5-Bromo-4-chloro-3-indolyl-phosphate (BCIP) staining is a simple and effective way to measure acid phosphatase (APase) activity on the root surface, phosphorus-starvation significantly increased the level of BCIP staining (Fig. 5d). The qRT-PCR results showed that the transcripts of *BrACP5* increased under phosphorus-deficient treatment (Fig. 5e). The above results suggest that *BrACP5* may regulate *B. rapa* leaf lobe formation through the phosphate-responsive pathway.

## Discussion

In this work, we applied a forward genetic approach to identify candidate genes underlying QTL controlling leaf lobes in *B. rapa. BrRCO* was predicted to be the most promising candidate gene for leaf lobe formation. Subsequently, the function of *BrRCO* was validated by CRISPR/Cas9-mediated gene knockout assay. Furthermore, *BrRCO* was functionally verified by over-expression in *B. rapa.* Gene evolution analysis revealed that *BrRCO*, was generally under negative selection, as a core gene responsible for leaf lobe formation in *B. rapa* and *B. napus. *BrRCO**, as an HD-ZIP I transcription factor, regulates the formation of leaf lobes in *B. rapa* by directly binding to the promoter of *BrACP5* and then inhibiting its expression.

### *RCO*: a gene duplication originated from its ancestral paralogous *LMI1* in the Brassicaceae

Variations in leaf margins, which may be entire or elaborate to form serrations or lobes, account for much of the variations in leaf shape [1]. According to this criteria, RcBr and NIL^RcBr^ can be classified as the simple group, while the materials of *B. napus* and *B. juncea* utilized in our research can be divided into the dissected group. Previous inheritance tests agreed that the lobed-leaf trait is controlled by one incompletely dominant gene in *B. napus* [17, 18] and *B. juncea* [20].

*RCO* has evolved from its ancestral paralog *LMI1* in the Brassicaceae by duplication. *LMI1* serves as a floral regulator, suggesting that *RCO* function was acquired by neo-functionalization [10]. Normally, *A. thaliana* does not show lobed leaves; however, the simple leaf can become a more complex one by transgenic overexpression of *RCO* [13]. After transferring *LMI-*like genes originating from other species into *Arabidopsis*, the leaf margins of the transgenic plants all showed varying degrees of lobed leaves [32]. In *C. hirsuta*, the *RCO* gene is expressed at the leaf base, while *ChLMI1* is expressed at the leaf margin [13]. The *RCO* and *ChLMI1* proteins have equivalent functions, and the species-specific action of *RCO* in leaflet formation reflects diversification of gene expression from its homolog *ChLMI1*. It has been shown that there is a unique enhancer sequence on the promoters of the *RCO* and *LMI1* genes, which determines the location of expression of each gene in the leaf [33].

In addition, our previous studies proved that these two paralogous genes, *BnA10. RCO* and *BnA10.LMI1*, function separately and that the deletion of either gene can result in an education in lobed leaf complexity [18, 19]. In *B. juncea*, *BjA10. RCO*, which is homologous to *BnA10. RCO* in *B. napus,* encodes an HD-Zip transcription factor and is identified to be the target gene responsible for leaf lobe formation, while *BjLMI* could not be found within the confidence interval [20, 21]. Recently, Li *et al.* [16] revealed that *BrLMI1* positively regulates leaf lobe formation in nonheading Chinese cabbage, which was functionally validated by overexpressing the CDS of *BrLMI1* transformed into *Arabidopsis* Col-0 plants and VIGS. However, there is no variation detected in the coding or promoter regions for *BrRCO* between the two parental nonheading Chinese cabbage lines [16], which was the opposite of our results. These results suggest that in *B. rapa*, *BrLMI*, and *BrRCO* each have an independent function in the regulation of leaf lobe formation. Nevertheless, the interaction between *RCO* and *LMI* in *Brassica* crops is still a complex area that needs to be resolved in the future.

### *BrRCO,* as an HD-ZIP I transcription factor regulates the leaf lobe formation in *B. rapa*

Several studies have demonstrated that leaf lobes in *B. rapa* (*AA*), *B. napus* (*AACC*), and *B. juncea* (*AABB)* are regulated by the major genes, *A10. RCO,* located in a hotspot region of chromosome A10, responsible for leaf lobe formation in *Brassica* crops. In our previous study, we developed an NIL^RcBr^ population using a lobed-leaf RcBr (*B. rapa* L. ssp. *dichotoma*) as the recurrent parent, and the entire-leaf Chinese cabbage inbred line, 08A061 (*B. rapa* L. ssp. *pekinensis*) as the donor parent [15], with a QTL *lob10.1* located at the end of chromosome A10. In this study, *lob10.1* was narrowed down to the range of 69.8 Kb on chromosome A10 in *B. rapa*, between two markers, A10–26 and A10–28 (Figs 1a and b and S1). Moreover, previous reports indicated that numerous variations have been identified in the regulatory regions of *BnA10. LMI*, *BnA10.RCO* and *BrA10. LMI*, in the coding sequence; however, no significant sequence variations were detected [16, 18, 19]. In our study, comparative allele sequencing showed that the main difference in coding sequence between the *RCO* gene in RcBr (lobed leaves) and NIL^RcBr^ (rounded/entire leaves) was a deletion of one base on the second exon in the NIL^RcBr^ allele, which ultimately leads to a frameshift (Fig. 1d and e and Figs S2–S5).

Stable genetic transformation is still challenging in *B. rapa* despite the small number of successful reports [34]. To study the function of candidate genes in *B. rapa*, most studies have used ectopic expression in *Arabidopsis* mutants, as well as transient transformation (VIGS). In *B. rapa*, many studies have predicted *BrRCO* as a leaf cleavage candidate gene; however, its function has yet to be validated [14, 15]. In this study, we successfully generated a *rco* mutant in RcBr and NIL^RcBr^ OE lines by cotyledon-mediated *Agrobacterium* infection, although only one T_0_ plant survived (Fig. 2). We also demonstrated that *BrRCO* can positively regulate leaf lobes in *B. rapa* using a combination with the T_1_, T_2_ phenotype, and Hi-Tom analysis (Figs 3 and S7). Hence, it is plausible to posit that *BrRCO*, as an HD-ZIP I transcription factor regulates the leaf lobe formation in *B. rapa*.

### *A10. RCO, a* negatively selected core gene controlling leaf lobe formation in Brassicaceae

In our previous study, we have shown that *BnA10. RCO* regulates leaf lobe formation by localization and CRISPR/Cas9 [19]. In this study, *BrRCO* controlling leaf lobe formation were functionally validated in *B. rapa* (Figs 2 and 3). Subsequently, the pangenome search confirmed that *A10. RCO* is a core gene in *B. rapa* and *B. napus*. In contrast, this gene was classified as variable in the *B. oleracea* pangenome. The presence–absence-status of this gene cannot be determined in *B. juncea* as there are no available pangenome assemblies for this species. Evolutionary analysis indicated that *A10. RCO* is negatively selected in *B. rapa* and *B. napus.* This is consistent with the phenotypic observation across diverse collections of *B. rapa* and *B. napus* lines, which showed that the majority of these lines have unlobed leaves. The synonymous substitution rate (ds) in *B. juncea* was negligible (almost zero) but significantly lower than the nonsynonymous mutation rate; thus, *A10. RCO* might be undergoing positive selection in this species. This coincides with the observation that almost all *B. juncea* lines showed lobed leaves (Tables S4–S6).

Promoter variations of *A10*. *LMI1* underlying leaf lobe formation were also functionally validated in *B. napus* by transformation [18] and *B. rapa* by ectopic expression in *Arabidopsis* [16]*.* Phylogenetic analysis revealed that *B. nigra* (*BB*) and *B. carinata* (*BBCC*) homologs were separated from the rest of the species, while *BrRCO* was clustered along with its *B. juncea*, *B. napus,* and pangenome homologs (Fig. 1f). This suggests that *BrRCO* may have followed a similar evolutionary trajectory in *B. juncea*, *B. napus*, and *B. oleracea*, which is likely influenced by the widespread domestication of these species.

### *BrRCO*-mediated leaf lobe formation by negatively regulated *BrACP5*

*RCO*, which encodes for a growth repressor, inhibits growth at the boundaries between leaflets/lobes. It was shown that *RCO* directly regulates the homeostasis of the hormone cytokinin to suppress growth at the leaf base [26]. Cytokinins play important roles in growth and development, directly controlling shoot meristem activity [24]. Furthermore, a multidirectional cross-regulation was observed between cytokinin, sugar, and phosphorus-starvation signaling [27]. In this study, a statistically significant enrichment of polysaccharide-responsive pathways was observed in the GO analysis of the DEGs, which is consistent with this view (Fig. S8).

In the present study, we used forward genetic analysis to identify *BrRCO* and have provided compelling evidence of its biological function in leaf lobe development. Multiple interaction tests verified that BrRCO targets the promoter of *BrACP5* (Fig. 4). Furthermore, the *ACP5*, induced by phosphorus-starvation in *Arabidopsis*, was identified as the target gene of BrRCO, and BrRCO inhibited the transcription level of *BrACP5*. The *acp5* single knockout plants by VIGS showed an absence of leaf lobe with the first two leaves (Figs 5a-b and S11).

Phosphorus serves as one of the three essential macronutrients for plants, and phosphate limitation leads to both molecular and developmental adaptations in all organisms. Starvation-responsive genes for phosphate includes those involved in cell biogenesis, cell division, protein synthesis, secondary metabolism, and stress response [35]. APase is a class of hydrolytic enzymes that degrade organic phosphorus to inorganic phosphorus, and phosphorus starvation induces APase gene expression [36]. Consistent with previous studies, we found that phosphorus starvation induces BrACP5 gene expression and enhances APase activity on the root surface of plants. In addition, we found that phosphorus deficiency caused the disappearance of leaf lobe (Fig. 5c-e).

Overall, our findings present a BrRCO-*BrACP5* model of lobe-leaf development driven by the phosphate-responsive pathway. However, in this study, *BrACP5* is a down-regulated gene identified between NIL^RcBr^ vs. rco and RcBr, as such it will be interesting to fully resolve the interactions between these two genes. Collectively, *BrRCO* may function as a negative regulator in the phosphate-responsive pathway, by directly binding to the upstream promoter regions of *BrACP5*, which needs to be further investigated.

## Experimental procedures

### Plant materials and growth conditions

All plants used in this study were *Brassica* crops. Initially, a set of NIL^RcBr^ was developed by four cycles of marker-assisted backcrossing followed by two cycles of selfing. Compared with the recurrent parent, RcBr, NIL^RcBr^ showed the absence of leaf lobes, while other agriculturally important traits showed no significant differences [15].

NIL^RcBr^ (female parent), RcBr (male parent), and their NIL^RcBr^-F_2_ (674 individuals) were cultivated in the Experiment Station of Shenyang Agricultural University, Shenyang, China (41.8°N, 123.4°E) in the autumn of 2017, and the F_2:3_ population of 22 families (4000 individuals) were derived by self-pollinating each F_2_ recombinant individual, respectively.

RcBr and NIL^RcBr^ were used as the transformation receptors in this study. All plants were grown at 25°C in the light incubator. Seeds were grown on Murashige and Skoog (MS) medium for four days, and grown with a 16/8-h light/dark photoperiod.

### Fine mapping of *lob10.1* and marker development

To narrow down the confidence interval underlying *lob10.1*, a larger NIL^RcBr^-F_2_ population of 674 individuals was used. Recombinants were screened using the markers W-InDel17 and W-InDel76 (Table S1), resulting in a total of 22 out of 674 recombinant individuals. In April 2019, the F_2:3_ populations consisting of 4000 individuals were sown by selfing the leaf lobed recombinant plants in the F_2_ families.

Molecular markers, including InDels and SNPs in the confidence interval of *lob10.1*, were newly developed based on RNA-Seq and whole-genome resequencing data analysis of the two parental lines (Table S1). Then, the recombinant individual plants of the F_2:3_ populations were further identified by KASP genotyping (Fig. S1) and PGE.

### Candidate gene prediction and cloning

To identify the candidate gene underpinning *lob10.1*, the sequence and annotation of the nine genes in the confidence interval were analyzed. Sequence variations between NIL^RcBr^ and RcBr were identified based on the whole-genome resequencing data of 15G. Gene annotation information in the confidence region was obtained from the *Brassica* database (BRAD; http://brassicadb.cn/#/Download/) and the *Arabidopsis* Information Resource (TAIR; http://www.arabidopsis.org/).

The HD ZIP I transcription factor *BrRCO* and *BrLMI* was finally predicted to be the candidate gene for *lob10.1*. The gDNA and cDNA of *BrLMI1*, *BrLMI2*, and *BrRCO* in NIL^RcBr^ and RcBr have been cloned for sequencing, respectively. Analysis of multiple sequence alignments was performed using the Sequencher 5.4.5 software.

### Phylogenetic analysis

The homolog of *BraA10g032440.3C* was searched across the *Brassica* genome references [37], including the pangenomes. The coding sequence of these homologs was utilized for the construction of a phylogenetic tree. The sequences were aligned using MUSCLE [38], which was used to generate a tree using RAxML with 1000 bootstraps. The best scoring tree was then visualized in the Geneious Prime software.

### Analysis of evolutionary selection

The coding sequence of *BraA10g032440.3C* was pairwise aligned with its homologs in the other *Brassica* species using MUSCLE [38]. The nonsynonymous (dN) and synonymous (dS) analyses were then performed on MEGAX software using the Jukes–Cantor method. The ratio was determined by dividing dN with dS.

### Homolog search in *Brassica* pangenomes

The full gene sequence of *BraA10g032440.3C* was BLAST-searched in the three *Brassica* pangenomes (*B. napus*, *B. oleracea*, and *B. rapa*) [28] with only the top hit considered as the likely homologous gene. The presence–absence status of the gene was then determined in each of the pangenomes (http://www.brassicagenome.net/databases.php).

### RNA extraction and qRT-PCR analysis

Total RNA was extracted from the shoot apex of seven day old parental lines (Fig. S12), NIL^RcBr^, rco mutants (by selfing the transgenic plant *rco-7-2*), and RcBr for RNA-seq analysis. For each sample, three biological replicates were conducted. RNA-seq was undertaken using the HiSeq 2000 platform (Illumina) by Personalbio (Shanghai, China). The raw data of RcBr and NIL^RcBr^ were mapped to the *B. rapa* reference genome from BRAD using Hisat2 (http://ccb.jhu.edu/software/hisat2/index.shtml). Genes that showed significant differential expression were selected for further analysis (>two-fold, i.e., log2-fold change >1 or log2-fold change <−1, padj <0.05).

qRT-PCR was used to determine the expression level of the target gene. Total RNA of VIGS-ACP5^RcBr^, NIL^RcBr^, and RcBr from the leaves were isolated using an RNA extraction kit (Aidlab Biotechnologies Co., Ltd., Beijing, China). A 1 μg sample of RNA was converted into cDNA following the manufacturer’s instructions (HiScript® III 1st Strand cDNA Synthesis Kit, Vazyme Biotech). RT-PCR was performed using the TransStart Top Green qPCR.

SuperMix Kit (TransGen Biotech) on a QuantStudio™ Real-Time PCR System (Thermo Fisher). Gene-specific primers were designed using Primer Premier 5.0 (Table S1), and the *Actin* gene was used as a reference for transcript level normalization [39].

### Subcellular localization of *BrRCO*

Full-length CDS of *BrRCO* without the stop codon was inserted into the binary vector pGADT7 to create the CaMV 35S: *BrRCO*-eGFP construct. The constructs were transiently transformed into tobacco (*Nicotiana benthamiana*) leaf epidermal cells by *Agrobacterium* (*A. tumefaciens*, GV3101)-mediated transformation. The GFP fluorescence was observed two days after transfection using a laser scanning confocal microscope (Nikon C2-ER, Nikon, Japan).

### Transformation and identification of mutants

Gene editing was performed using the binary pYLCRIPSR/Cas9 multiplex genome-targeting vector system [40]. Sequence-specific sgRNAs selection in the target gene and CRISPR/Cas9 construct assembly was carried out as per our previous study [19] . The construct was transformed into RcBr [41] via the *A. tumefaciens*-mediated cotyledon method.

The full *RCO^RcBr^* CDS was amplified from RcBr shoot apex cDNA to generate the *RCO*^RcBr^-OE construct pBWA(V)HS-RCO^RcBr^-OE. *A. tumefaciens*-mediated cotyledon transformation was used to transform the construct into NIL^RcBr^.

All positive plants were screened using PCR to amplify genomic DNA. Then, the Hi-TOM method was used to determine the target mutations in transgenic plants as described by Zhai *et al.* [42]. Primers for the specific detection of the target are listed in Table S1.

### DAP-seq

Two technical replicates, OVHY-1 and OVHY-2, as well as input (vector PT7SP6) control DAP-seq libraries were constructed. The clean reads were aligned to the *Brassica rapa*_V3.0 reference genome [35] using the software Bowtie 2 version 2.3.4.3 set to default parameters [43]. The Model-based Analysis of ChIP-Seq algorithm (MACS) version 2.2.7.1 was conducted to detect peaks with BAMPE mode [44]. The target genes of the *BrRCO*^RcBr^ was conserved motifs in the peak region were analyzed using the MEME software [45].

### Y1H assay

To validate the target genes of *BrRCO*, the full-length of *RCO*^RcBr^-CDS were cloned into the pGADT7 plasmid. The −624 to −274 (AAATAATT) sequence upstream of the start site of translation initiation of *BrSLOMO* (*SLOW MOTION*), +68 to +560 (AAATAATT) sequence downstream of the translation initiation start site of *BrACP5* (*ACYL CARRIER PROTEIN 5*) were cloned into the pAbAi plasmid. The p53-AbAi, pBrSLOMO-AbAi, and pBrACP5-AbAi competent cells were prepared separately. Subsequently, pGADT7 was transformed into the p53-AbAi competent cells as a positive control and pGADT7 was transformed into pBrSLOMO-AbAi/pBrACP5-AbAi competent cells as a negative control. After PCR, the positive colony was cultured on a medium without uracil (Ura) and leucine (Leu) (Simple Dropout/-Ura/-Leu) with no AbA and then on a medium without Ura and Leu with 100 ng/ml ABA (Simple Dropout/-Ura/-Leu/+AbA).

### LUC assay

The CDS without the stop codon of *BrRCO*^RcBr^ was cloned into the pGreenII-62SK vector, and the CDS of *BrACP5* were separately cloned into pGreenII 0800-LUC vectors. Grouped vectors were transiently co-transformed into *N. benthamiana* leaves by *Agrobacterium* infiltration [46]. The luciferase activities were analyzed 48 h after infiltration using the Thermo Sorvall ST16R (Thermo Scientific).

### EMSA

Full-length *BrRCO* CDS fused with His-tag was cloned into the pET30a vector, introduced into *Escherichia coli* BL21, and colony PCR was performed, then incubated with 0.2 mM IPTG (Isopropyl β-D-Thiogalactoside) for 16 h. Two 5′-biotinylated oligonucleotides (5'-TTAATCAAATAATTCTGATT-3′; 5′-AATTTTGATTATTATAGATG-3′) were used as the probes (Fig. S13). Probes were labeled with His-A, and then the unlabeled probe was used as a competitor. The probes were incubated with nuclear extract at room temperature for 30 min. The entire reaction mixture was run on a non-denaturing 6% polyacrylamide gel (0.5 × TBE, time: 1 h, volts: 80 V, temp:4°C), and then transferred onto Biodyne® B nylon membranes (Pall Corporation). Signals were visualized using kit reagents and ChemiDoc XRS [47] (BioFGG-Rad Laboratories, USA).

### TRV-based VIGS assay

To construct the VIGS vector of *BrACP5*, a sequence-specific cDNA in the target gene was selected using the web-based tool Sol Genetics Network VIGS (https://vigs.solgenomics.net/). This cDNA fragment was then cloned into pTRV2 to construct pTRV2-ACP5. VIGS-ACP5 plants were obtained by silencing *BrACP5* in RcBr background. The VIGS vectors pTRV1 and pTRV2 and the protocol for the VIGS assay have been presented previously [48].

### Phosphorus depletion experiment

The RcBr was used for all physiological experiments. Hydroponic experiments were conducted using a modified Hoagland’s culture solution, as a phosphorus-deficient condition, containing the nutrients necessary for the growth of cabbage except for phosphorus. Plants were grown at 25°C in a light incubator for six weeks and treated with 0, 50, 100, or 150 mg/L phosphorus.

### 5-Bromo-4-chloro-3-indolyl-phosphate staining

Seeds were germinated on +P and -P nutrient solutions and grown for six weeks. The roots of seedlings were excised and incubated on a 5-bromo-4-chloro-3-indolylphosphate–agar solution containing 10 mM phosphate buffer (pH 5.7), 0.5% agar and 0.01% BCIP for 12 h at 25°C. Cleavage of BCIP by APase produces blue precipitates, which were imaged [49].

### Accession numbers

Sequence data from this article can be found in the GenBank data libraries under accession numbers PRJNA1068137 (Re-sequencing of RcBr and NIL^RcBr^), PRJNA1070011 (RNA-seq of *rco-7-2* and RcBr), PRJNA1069989 (RNA-seq of NIL^RcBr^ and RcBr), PRJNA1077467 (DAP-seq of *BrRCO*^RcBr^), and PRJNA1078095 (Hitom analysis of *Br-rco* mutants). The following materials are available in the online version of this article**.**

## Supplementary Material

Web_Material_uhaf084

## Data Availability

The paper contains the data supporting the findings of this study, and supplementary data is available online.
