# Supplementary material for: BrRCO promotes leaf lobe formation by repressing BrACP5 expression in Brassica rapa
Source: Hortic Res. 2025 Mar 12;12(5):uhaf084. doi: 10.1093/hr/uhaf084 (PMC11986587; doi:10.1093/hr/uhaf084)
Supplement: Web_Material_uhaf084 [file web_material_uhaf084.zip › Supplemental figures-.pdf]

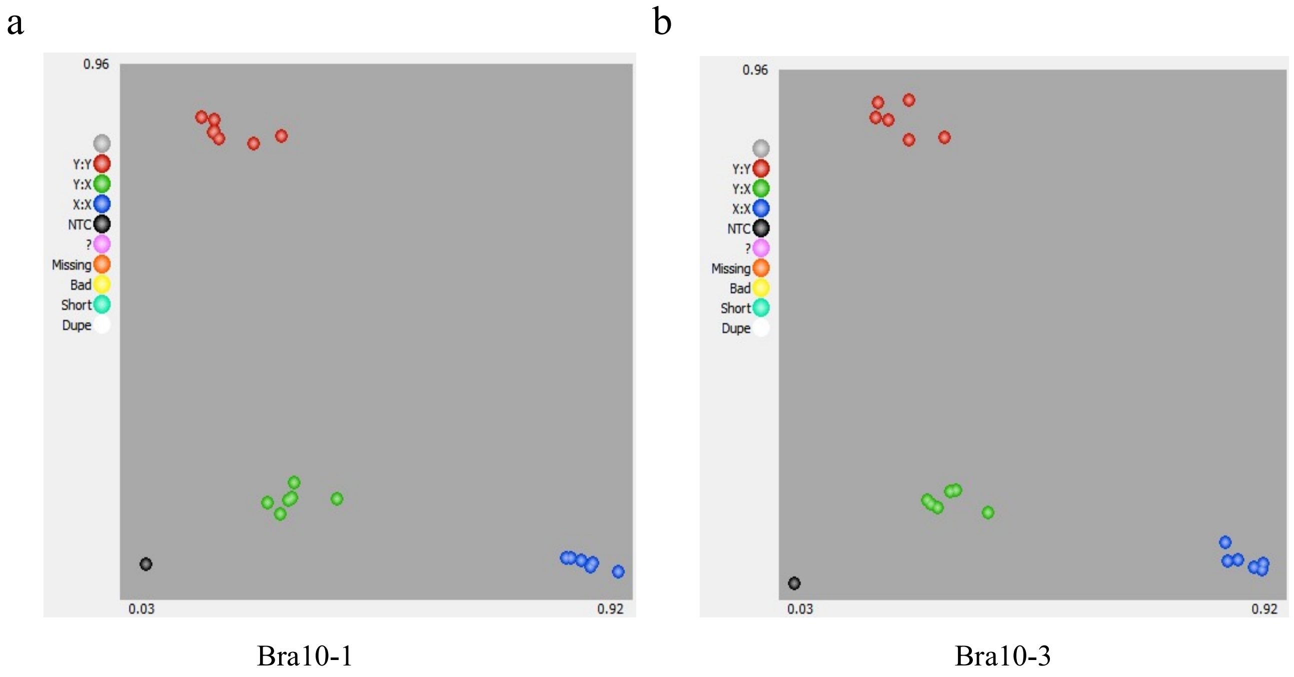

Figure S1. Primer polymorphism screening by KASP genotyping.
The red ball represents the NIL^{RcBr} homozygous genotype, the blue ball represents the RcBr homozygous genotype, the green ball represents the heterozygous genotype, while black ball as NTC.

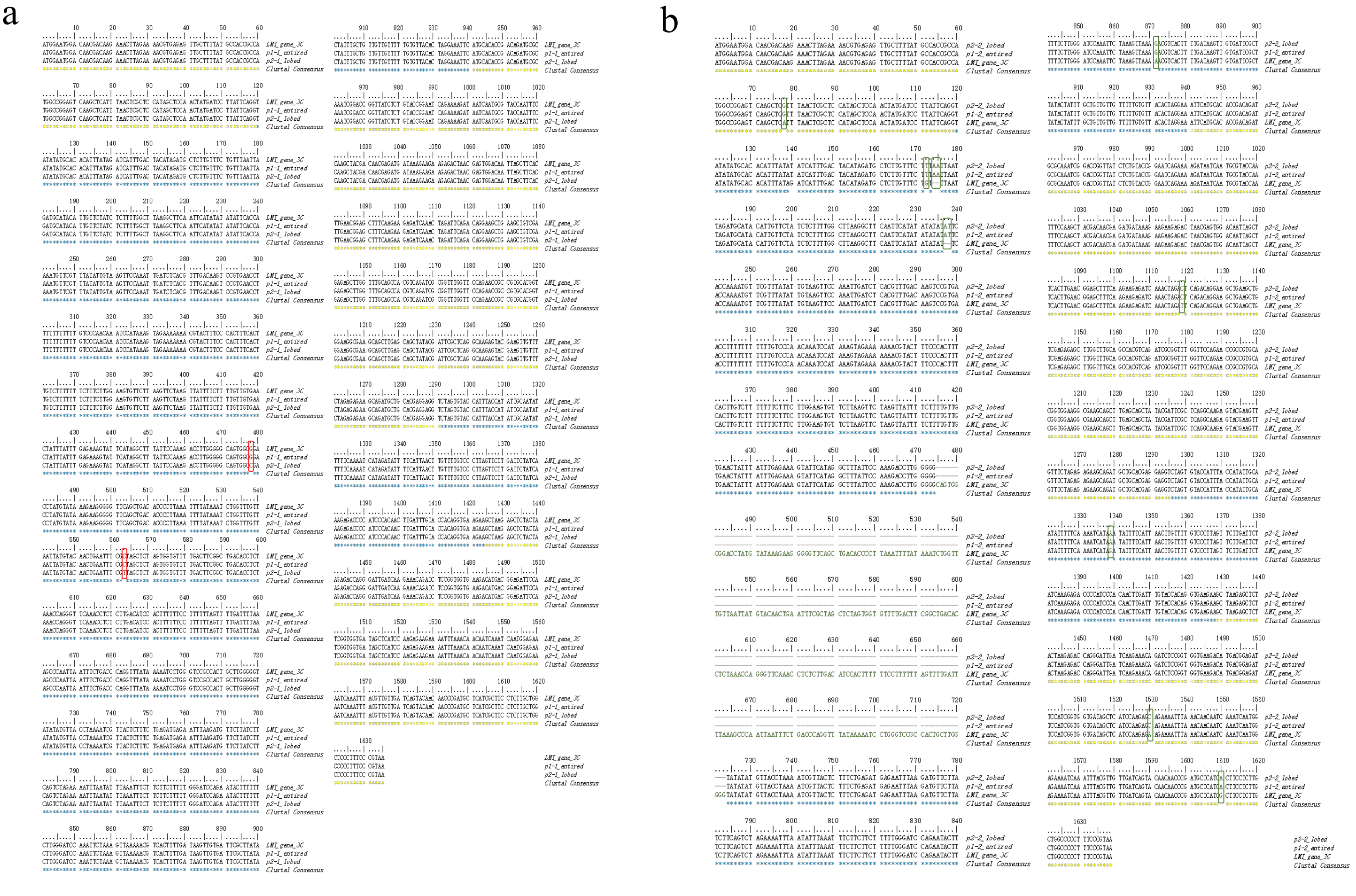

Figure S2. Squencing comparison between NIL^{RcBr} and RcBr for *BrLMI-1* (a)/*BrLMI-2* (b). The reference genome sequence is version 3.0. There are two SNPs between NIL^{RcBr}(P1) and RcBr(P2) located in the intron region of *BrLMI-1*(a) marked with a red box. Green box indicates the location of the differences between two parents and the reference genome of *BrLMI-2*(b). P1:NIL^{RcBr}, P2:RcBr.

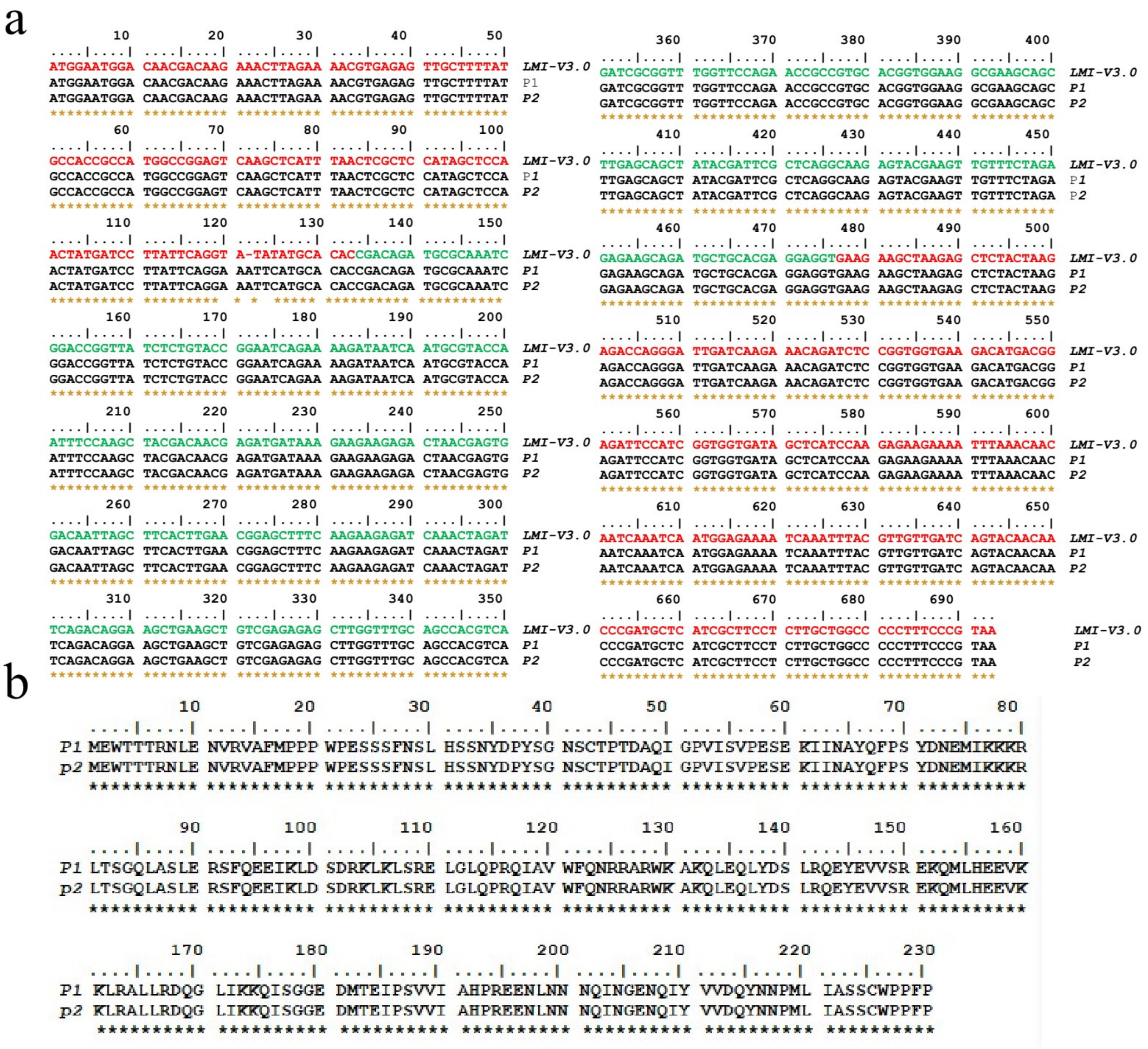

Figure S3. cDNA cloning sequencing comparison results of *BrLMI*(a) and Protein sequence prediction(b).

There are three exons of *BrLMI* separate by color. No differences found. P1:NIL[RcBr], P2:RcBr.

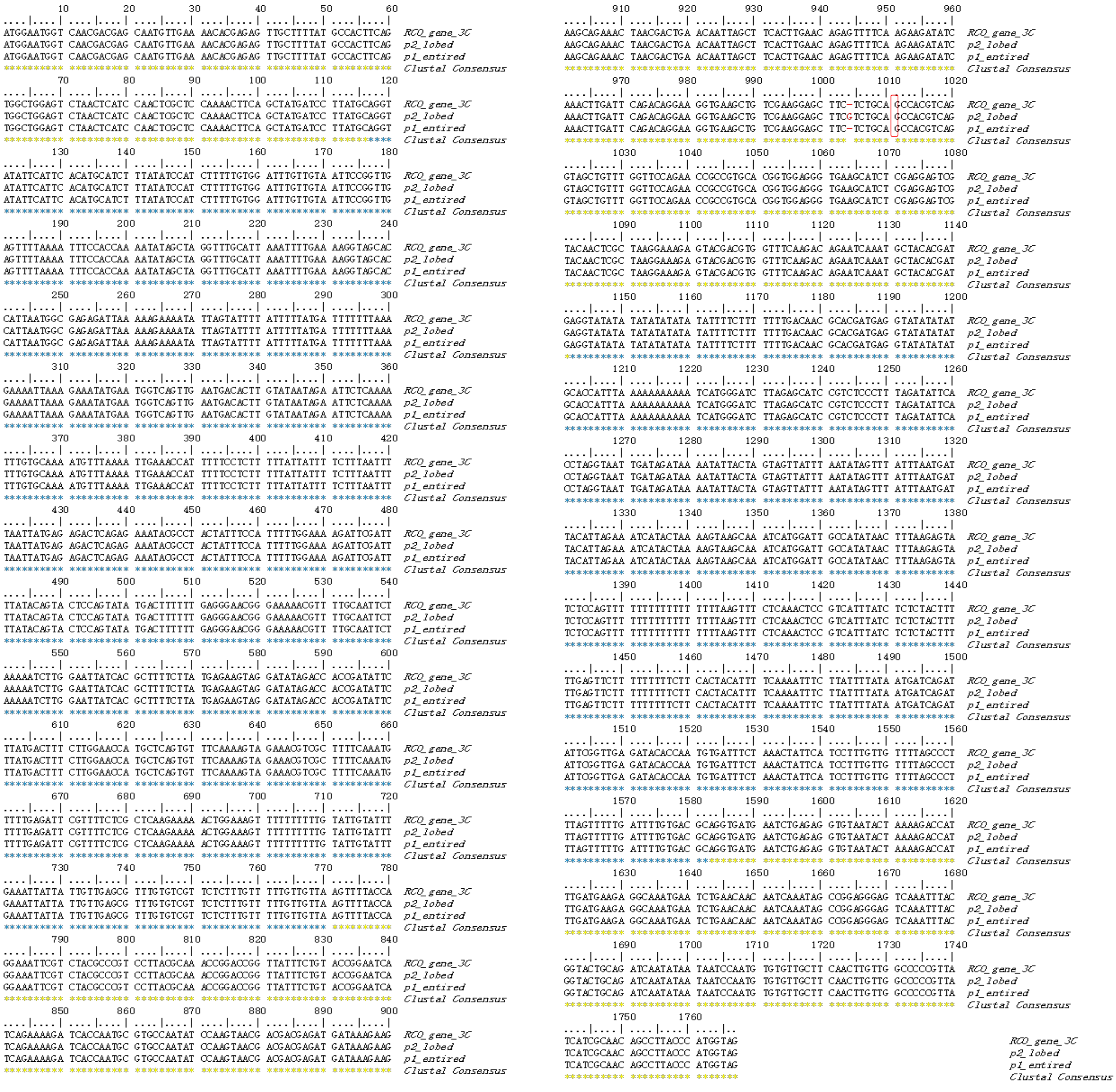

Figure S4. Gene cloning sequencing comparison results of *BrRCO*.

The difference between NIL^{RcBr} and RcBr marked with a red box. P1:NIL^{RcBr}, P2:RcBr.

```
         10         20         30         40         50
....|....| ....|....| ....|....| ....|....| ....|....|
ATGGAATGGT CAACGACGAG CAATGTTGAA AACACGAGAG TTGCTTTTAT  RCO-V3.0
ATGGAATGGT CAACGACGAG CAATGTTGAA AACACGAGAG TTGCTTTTAT  P1
ATGGAATGGT CAACGACGAG CAATGTTGAA AACACGAGAG TTGCTTTTAT  P2
********** ********** ********** ********** **********
         60         70         80         90        100
....|....| ....|....| ....|....| ....|....| ....|....|
GCCACTTCAG TGGCTGGAGT CTAACTCATC CAACTCGCTC CAAAACTTCA  RCO-V3.0
GCCACTTCAG TGGCTGGAGT CTAACTCATC CAACTCGCTC CAAAACTTCA  P1
GCCACTTCAG TGGCTGGAGT CTAACTCATC CAACTCGCTC CAAAACTTCA  P2
********** ********** ********** ********** **********
        110        120        130        140        150
....|....| ....|....| ....|....| ....|....| ....|....|
GCTATGATCC TTATGCAGTT TTACCAGGAA ATTCGTCTAC GCCCGTCCTT  RCO-V3.0
GCTATGATCC TTATGCAGTT TTACCAGGAA ATTCGTCTAC GCCCGTCCTT  P1
GCTATGATCC TTATGCAGTT TTACCAGGAA ATTCGTCTAC GCCCGTCCTT  P2
********** ********** ********** ********** **********
        160        170        180        190        200
....|....| ....|....| ....|....| ....|....| ....|....|
ACGCAAACCG GACCGGTTAT TTCTGTACCG GAATCATCAG AAAAGATCAC  RCO-V3.0
ACGCAAACCG GACCGGTTAT TTCTGTACCG GAATCATCAG AAAAGATCAC  P1
ACGCAAACCG GACCGGTTAT TTCTGTACCG GAATCATCAG AAAAGATCAC  P2
********** ********** ********** ********** **********
        210        220        230        240        250
....|....| ....|....| ....|....| ....|....| ....|....|
CAATGCGTGC CAATATCCAA GTAACGACGA CGAGATGATA AAGAAGAAGC  RCO-V3.0
CAATGCGTGC CAATATCCAA GTAACGACGA CGAGATGATA AAGAAGAAGC  P1
CAATGCGTGC CAATATCCAA GTAACGACGA CGAGATGATA AAGAAGAAGC  P2
********** ********** ********** ********** **********
        260        270        280        290        300
....|....| ....|....| ....|....| ....|....| ....|....|
AGAAACTAAC GACTGAACAA TTAGCTTCAC TTGAACAGAG TTTTCAAGAA  RCO-V3.0
AGAAACTAAC GACTGAACAA TTAGCTTCAC TTGAACAGAG TTTTCAAGAA  P1
AGAAACTAAC GACTGAACAA TTAGCTTCAC TTGAACAGAG TTTTCAAGAA  P2
********** ********** ********** ********** **********
        310        320        330        340        350
....|....| ....|....| ....|....| ....|....| ....|....|
GATATCAAAC TTGATTCAGA CAGGAAGGTG AAGCTGTCGA AGGAGCTTC-  RCO-V3.0
GATATCAAAC TTGATTCAGA CAGGAAGGTG AAGCTGTCGA AGGAGCTTC-  P1
GATATCAAAC TTGATTCAGA CAGGAAGGTG AAGCTGTCGA AGGAGCTTCG  P2
********** ********** ********** ********** *********

        360        370        380        390        400
....|....| ....|....| ....|....| ....|....| ....|....|
TCTGCAGCCA CGTCAGGTAG CTGTTTGGTT CCAGAACCGC CGTGCACGGT  RCO-V3.0
TCTGCAGCCA CGTCAGGTAG CTGTTTGGTT CCAGAACCGC CGTGCACGGT  P1
TCTGCAGCCA CGTCAGGTAG CTGTTTGGTT CCAGAACCGC CGTGCACGGT  P2
********** ********** ********** ********** **********
        410        420        430        440        450
....|....| ....|....| ....|....| ....|....| ....|....|
GGAGGGTGAA GCATCTCGAG GAGTCGTACA ACTCGCTAAG GAAAGAGTAC  RCO-V3.0
GGAGGGTGAA GCATCTCGAG GAGTCGTACA ACTCGCTAAG GAAAGAGTAC  P1
GGAGGGTGAA GCATCTCGAG GAGTCGTACA ACTCGCTAAG GAAAGAGTAC  P2
********** ********** ********** ********** **********
        460        470        480        490        500
....|....| ....|....| ....|....| ....|....| ....|....|
GACGTGGTTT CAAGACAGAA TCAAATGCTA CACGATGAGG TGATGAATCT  RCO-V3.0
GACGTGGTTT CAAGACAGAA TCAAATGCTA CACGATGAGG TGATGAATCT  P1
GACGTGGTTT CAAGACAGAA TCAAATGCTA CACGATGAGG TGATGAATCT  P2
********** ********** ********** ********** **********
        510        520        530        540        550
....|....| ....|....| ....|....| ....|....| ....|....|
GAGAGGTGTA ATACTAAAAG ACCATTTGAT GAAGAGGCAA ATGAATCTGA  RCO-V3.0
GAGAGGTGTA ATACTAAAAG ACCATTTGAT GAAGAGGCAA ATGAATCTGA  P1
GAGAGGTGTA ATACTAAAAG ACCATTTGAT GAAGAGGCAA ATGAATCTGA  P2
********** ********** ********** ********** **********
        560        570        580        590        600
....|....| ....|....| ....|....| ....|....| ....|....|
ACAACAATCA AATAGCCGGA GGGAGTCAAA TTTACGGTAC TGCAGATCAA  RCO-V3.0
ACAACAATCA AATAGCCGGA GGGAGTCAAA TTTACGGTAC TGCAGATCAA  P1
ACAACAATCA AATAGCCGGA GGGAGTCAAA TTTACGGTAC TGCAGATCAA  P2
********** ********** ********** ********** **********

        610        620        630        640        650
....|....| ....|....| ....|....| ....|....| ....|....|
TATAATAATC CAATGTGTGT TGCTTCAACT TGTTGGCCCC CGTTATCATC  RCO-V3.0
TATAATAATC CAATGTGTGT TGCTTCAACT TGTTGGCCCC CGTTATCATC  P1
TATAATAATC CAATGTGTGT TGCTTCAACT TGTTGGCCCC CGTTATCATC  P2
********** ********** ********** ********** **********
        660        670
....|....| ....|....| ..
GCAACAGCCT TACCCATGGT AG                                RCO-V3.0
GCAACAGCCT TACCCATGGT AG                                P1
GCAACAGCCT TACCCATGGT AG                                P2
********** ********** **
```

Figure S5. cDNA cloning sequencing comparison results of *BrRCO*.

There are three exons of *BrRCO* separate by different color. A G-Base insertion located in the second exon region marked with black box. P1:NIL^{RcBr}, P2:RcBr.

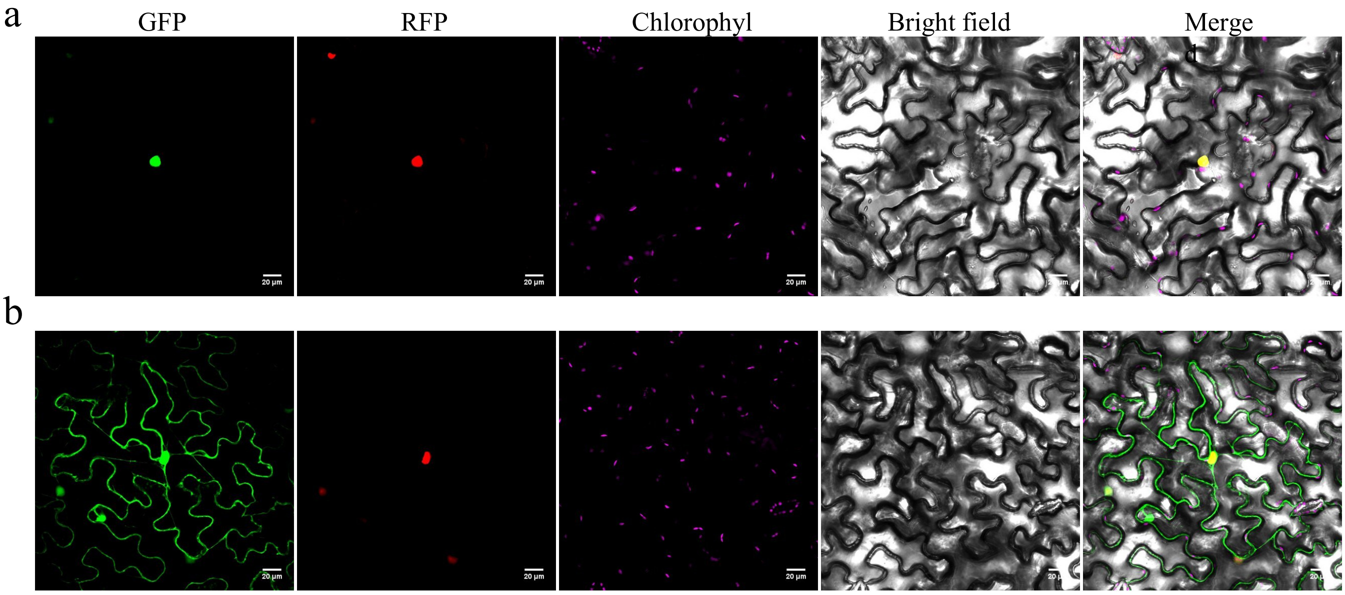

Figure S6. Subcellular localization analysis of *BrRCO*. *Agrobacterium* cells carrying 35S:BrRCO-eGFP (a) or 35S:eGFP (b) construct were infiltrated into 4-week-old tobacco leaves. GFP signals were examined two days after agro-infiltration. The nuclear protein OsGHD7-fused RFP was used as a nuclear marker. Chlorophyll: auto fluorescence signals of chloroplasts. Bars = 20 μm.

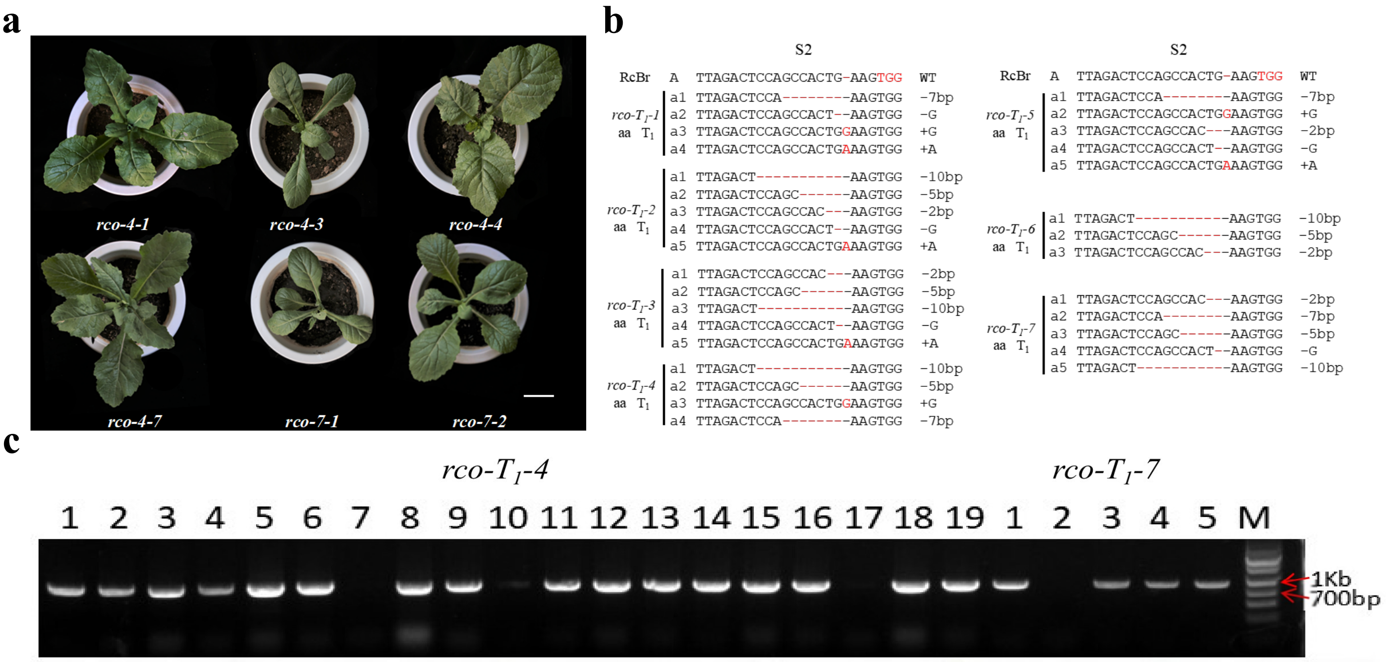

Figure S7. T_2 generation seedling phenotype of *rco-T_1-4* and *rco-T_1-7* lines and positive test results for the transgene.

(a) Phenotypes of the six T_2 monoclonal strains. Bars = 6 cm. (b) Six T_2 edit types of strains, corresponding to (a), respectively. The nucleotide InDels are marked in red, with details labeled at right; "A" represents the WT allele, while "a" represents the mutated allele. (c) The positive test of T_2 generation, with 19 self-crosses of *rco-T_1*-4 lineage and five self-crosses of *rco-T_1-7* lineage. M: marker.

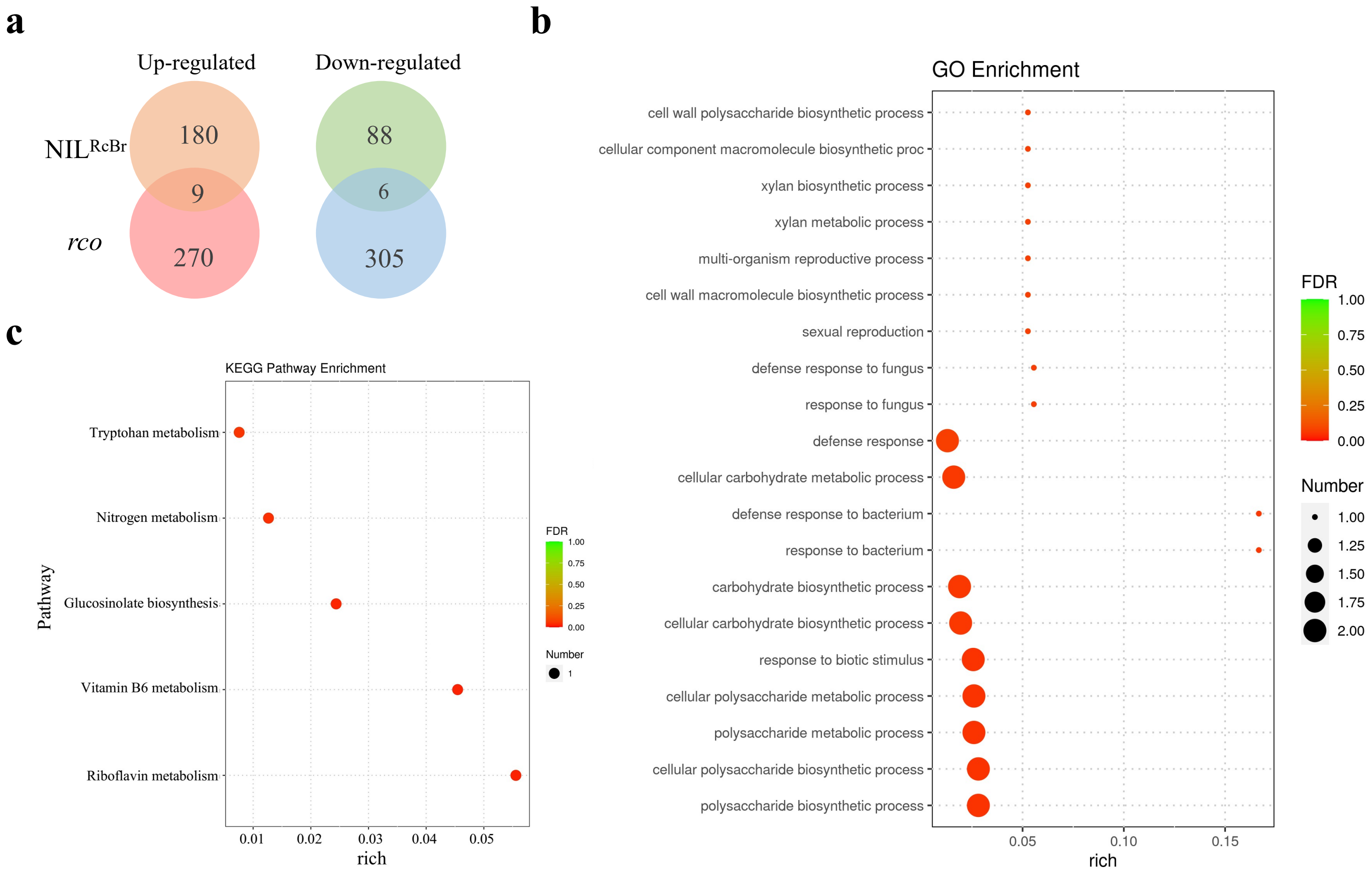

Figure S8. Identification of genome-wide direct targets of BrRCO.
(a) Venn diagram of the DEGs of NIL^{RcBr} and *rco* mutant with RcBr. Accordingly, nine up-regulated and six down-regulated DEGs were identified between NIL^{RcBr} vs. RcBr and *rco* mutant vs. RcBr. (b,c) The top GO-enriched and KEGG-enriched terms of DEGs from RNA-seq of both NIL^{RcBr} vs. RcBr and *rco-7-2* vs. RcBr.

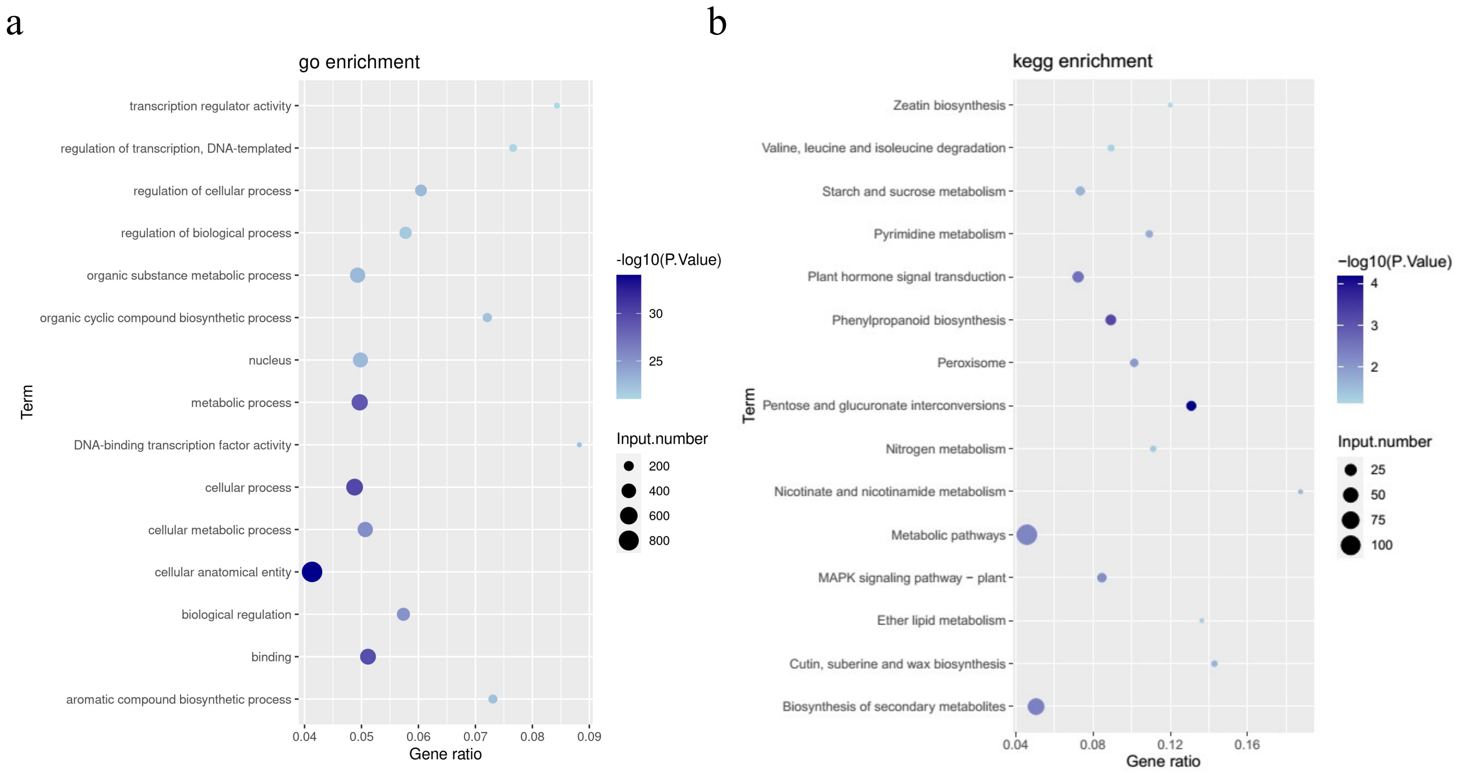

Figure S9. The top GO-enriched and KEGG-enriched terms of DAP-seq.

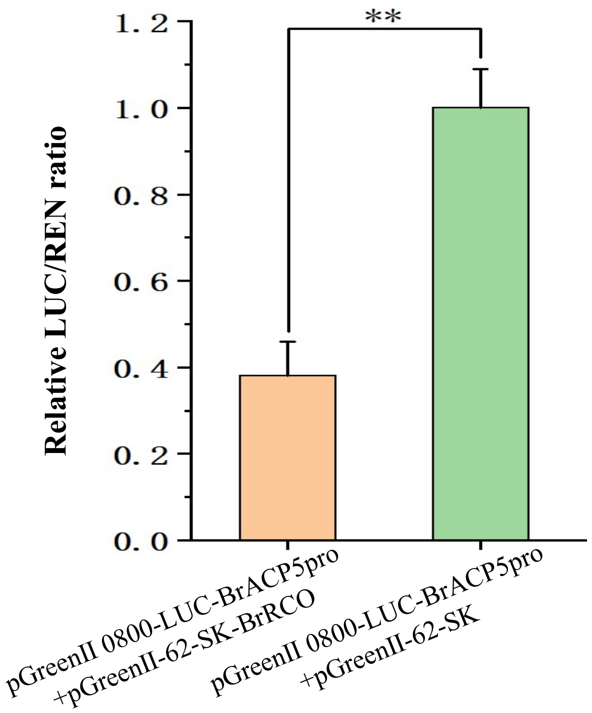

Figure S10. The LUC activity detection analysis.

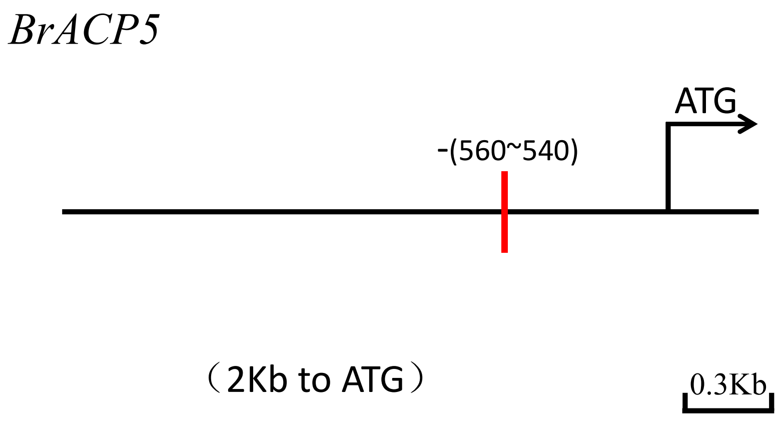

Figure S11. The probes used for EMSA analysis.
The red vertical line indicates the position of the probe in the promoter region.

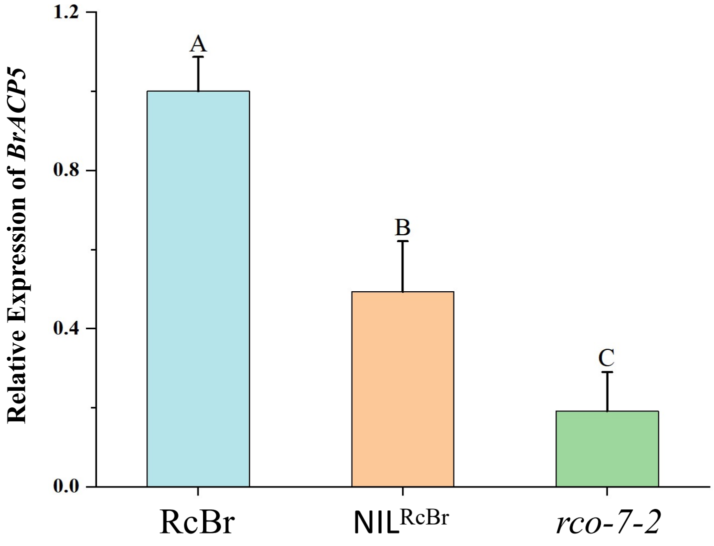

Figure S12. Expression analysis of the *BrACP5* gene in shoot apex of RcBr, NIL^{RcBr} and mutant *rco-7-2*.

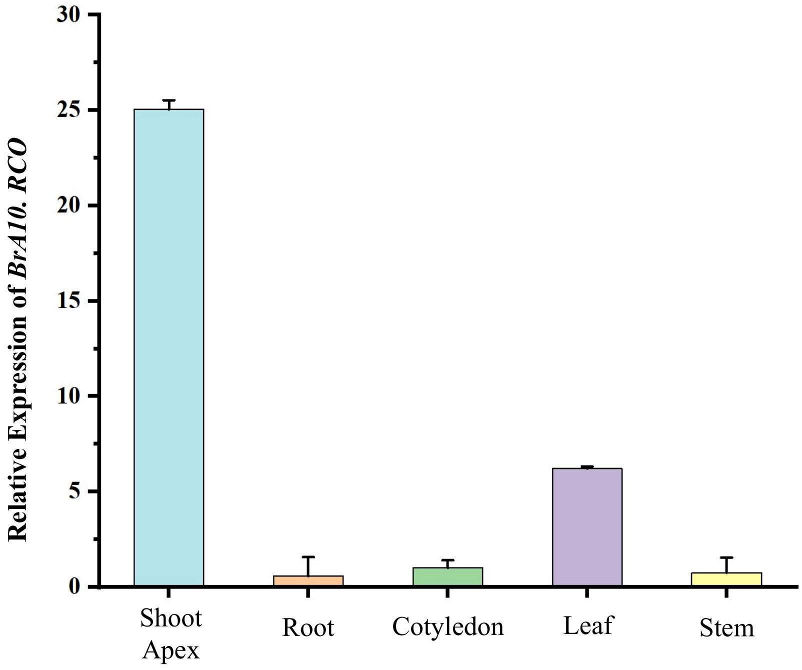

Figure S13. Expression analysis of candidate genes for the control of leaf lobing in RcBr.
